# Pyrolyzed Biomass Filler for PLA-Based Food Packaging

**DOI:** 10.3390/polym17101327

**Published:** 2025-05-13

**Authors:** Andreea-Cătălina Joe, Maria Tănase, Catalina Călin, Elena-Emilia Sîrbu, Ionuț Banu, Dorin Bomboș, Stanca Cuc

**Affiliations:** 1 Chemistry Department, Petroleum-Gas University of Ploiesti, 39 Bucharest Blvd., 100680 Ploiesti, Romania; andreea-catalina@upg-ploiesti.ro (A.-C.J.); catalina.calin20@yahoo.com (C.C.); elena.oprescu@upg-ploiesti.ro (E.-E.S.); 2 Mechanical Engineering Department, Petroleum-Gas University of Ploiesti, 39 Bucharest Blvd., 100680 Ploiesti, Romania; maria.tanase@upg-ploiesti.ro; 3 National Institute for Research & Development in Chemistry and Petrochemistry ICECHIM, 060021 Bucharest, Romania; 4 Department of Chemical and Biochemical Engineering, National University of Science and Technology POLITEHNICA Bucharest, 313 Spl. Indepentenței, 060042 Bucharest, Romania; 5 S.C. Medacril S.R.L., 8 Carpati Street, 551022 Medias, Romania; 6 Raluca Ripan Institute of Research in Chemistry, “Babes Bolyai” University, 400294 Cluj-Napoca, Romania; stanca.boboia@ubbcluj.ro

**Keywords:** PLA, composites, biochar, TGA, DSC, SEM, AFM

## Abstract

Poly(lactic acid) (PLA) is a biodegradable thermoplastic polymer used in various applications, including food packaging, 3D printing, textiles, and biomedical devices. Nevertheless, it presents several limitations, such as high hydrophobicity, low gas barrier properties, UV sensitivity, and brittleness. To overcome this issue, in this study, biochar (BC) produced through pyrolysis of bio-mass waste was incorporated (1 wt.%, 2wt.%, and 3 wt.%—PLA 1, PLA 2, and PLA 3) to enhance thermal and mechanical properties of PLA composites. The impact of pyrolysis temperature on the kinetic parameters, physicochemical characteristics, and structural properties of banana and orange peels for use as biochar added to PLA was investigated. The biomass waste such as banana and orange peels were characterized by proximal analysis and thermogravimetric analysis (TGA); meanwhile, the PLA composites were characterized by tensile straight, TGA, differential scanning calorimetry (DSC), scanning electron microscopy (SEM), and atomic force microscopy (AFM). The results indicated that the presence of biochar improved hygroscopic characteristics and Tg temperature from 62.98 °C for 1 wt.% to 80.29 °C for 3 wt.%. Additionally, it was found that the tensile strength of the composites increased by almost 30% for PLA 3 compared with PLA 1. The Young’s modulus also increased from 194.334 MPa for PLA1 to 388.314 MPa for PLA3. However, the elongation decreased from 14.179 (PLA 1) to 7.240 mm (PLA3), and the maximum thermal degradation temperature shifted to lower temperatures ranging from 366 °C for PLA-1 to 345 °C for PLA-3 samples, respectively. From surface analysis, it was observed that the surface of these samples was relatively smooth, but small microcluster BC aggregates were visible, especially for the PLA 3 composite. In conclusion, the incorporation of biochar into PLA is a promising method for enhancing material performance while maintaining environmental sustainability by recycling biomass waste.

## 1. Introduction

Biochar (BC) is typically produced through pyrolysis [1,2], a thermochemical process that decomposes organic material in an oxygen-limited environment [3]. Biochar (BC), a bio-derived carbon, is a promising alternative to traditional carbonaceous [4,5,6], non-renewable fossil-based fillers like carbon tubes, carbon black, carbon nanotubes, and graphene [7]. Feliz Florian *et al.* [8] provided a comprehensive review of biochar production and its potential applications in biocomposite materials, highlighting the influence of pyrolysis parameters such as temperature, heating rate, and feedstock type on biochar characteristics. Additionally, Alhelal *et al.* [9] explored the use of spent coffee ground-derived biochar in 3D-printed epoxy composites, demonstrating that at 1 wt.%, it enhanced mechanical properties, but higher loading (3 wt.%) reduced both viscoelastic and flexural properties of 3D-printed samples.

The work performed by Călin *et al.* [10] investigated the pyrolytic decomposition of apple and potato peel waste using TGA, FTIR, SEM, and EDS. It analyzed degradation kinetics and the impact of temperature (25–800 °C) on biochar properties. Results showed distinct thermal behaviors, with apple peel degrading in two stages and potato peel in one. Higher pyrolysis temperatures enhanced porosity and adsorption capacity, making the biochar effective for nitrate removal and a potential sustainable material.

Poly(lactic acid) (PLA) is a biodegradable thermoplastic derived from natural resources like corn, sugar cane, and beetroot [11,12,13] and is widely utilized in diverse applications, including food packaging, 3D printing filaments, textiles, and biomedical devices [14,15,16].

The incorporation of biochar into PLA has been studied extensively to improve its mechanical, thermal, and electrical properties. Ho *et al.* [17] investigated the enhancement of PLA properties using bamboo charcoal particles, reporting improved mechanical strength and thermal stability. The composites (2.5–10 wt.% BC) showed tensile, flexural, and ductility improvements of 43%, 99%, and 52%, respectively. However, higher BC content lowered the glass transition temperature. While compost degradation weakens mechanical properties, UV exposure has a lesser impact, highlighting BC/PLA’s potential for durable applications. Similarly, George *et al.* [18] demonstrated that coconut shell biochar obtained through the pyrolysis of coconut shell in an inert atmosphere enhances both electrical and mechanical properties of PLA/polybutylene adipate-co-terephthalate (PBAT) composites, making them suitable for antistatic applications. Vidakis *et al.* [19] explored the impact of biochar content on additive manufacturing, finding that increasing biochar concentration improved critical quality indicators such as dimensional stability and mechanical performance. The study compared the effects of biochar on five polymeric matrices—ABS, HDPE, PETG, PP, and PLA—in additive manufacturing. Quality metrics such as surface roughness, porosity, and dimensional accuracy were analyzed and correlated with mechanical performance. Among the composites, 4 wt.% biochar-loaded polymers exhibited the lowest porosity and dimensional deviations, achieving the highest tensile strength. Arrigo *et al.* [20] further examined the rheological and thermal properties of PLA–biochar composites, emphasizing how processing conditions and filler content influence final material characteristics. Rheological analysis confirmed effective filler dispersion but revealed PLA molar mass reduction at high temperatures. Melt-mixed composites showed altered rheology and dual melting peaks, while solvent-cast biocomposites exhibited increased crystallinity at low filler content. Thermogravimetric analysis indicated reduced thermal stability due to BC’s catalytic effect on PLA degradation.

The work of Zouari *et al.* [21] examined the impact of biochar (BC) on the mechanical, thermal, and water-resistance properties of PLA and hemp–PLA composites. Results showed that 5 wt.% BC improved tensile strength and modulus, while thermal stability slightly decreased due to BC’s catalytic effect. Higher BC content reduced water resistance due to its hydrophilicity and porosity.

Similarly, the study of Aup-Ngoen and Noipitak [22] explored the use of agricultural biomass residues (cassava rhizome, durian peel, pineapple peel, and corncob) for carbon-rich biochar (CRB) production via pyrolysis. Characterization revealed that carbon content depends on biomass origin, with cassava rhizome being the most suitable precursor. Higher pyrolysis temperatures increased CRB carbon content. PLA composites with high-carbon CRB showed enhanced mechanical properties, including improved elastic modulus and impact energy, highlighting CRB’s potential for sustainable PLA biocomposites.

Day *et al.* [23] examined the impact of biochar content and additives (polyethylene glycol, STRUKTOL^®^, and cork particles) on PLA composites. Cork-based composites exhibited higher tensile strength (51–54 MPa) than plasticizer-based composites (41–46 MPa). Scanning electron microscopy showed aggregation in the 10 wt.% biochar composites, with cork composites (1 and 3 wt.%) showing less aggregation.

Several studies have investigated the impact of biochar on PLA’s microstructure and processing behavior. Anerao *et al.* [24] conducted a parametric study on (rice husk) biochar-reinforced PLA composites for fused deposition modeling (FDM), and it was found that for 0.3 mm layer thickness, 5 wt.% biochar improved tensile strength (36 MPa) and modulus (1103 MPa) but reduced flexural strength. The highest flexural modulus was achieved with 3 wt.% biochar, 100% infill, and 0.1 mm layers. While 1 wt.% biochar increased impact strength, higher content led to agglomeration effects. Gazzotti *et al.* [25] examined cellulose nanofibrils as reinforcing agents in PLA composites, providing insights into nanoscale enhancements that complement biochar-based modifications. Additionally, Huang *et al.* [26] studied the effects of grapevine (GVC) biochar on PLA composites, demonstrating that the GVC/PLA composite achieved tensile and impact strengths of 79.79 MPa and 22.67 J/m, increasing by 41.4% and 32.1% over pristine PLA. However, PLA’s molecular weight decreased with higher GVC content. Other studies developed composite materials between PLA and an additive based on an aromatic carbodiimide for packaging use [27].

The citrus fruits industry, producing over 161 million tons in 2021, generates significant waste, including seeds, membranes, and peel, which comprises up to 60% the weight of fresh fruit. The peel is the most common waste reported by citrus processing industries [28]. Raimondo *et al.* [29] estimated that 31.2 million tons of citrus fruits are processed every year, with 50–60% of the initial mass ending as residue. The integration of orange peel powder into biobased matrices has drawn interest due to its potential applications. The second most produced fruit after citrus, comprising 16% of global fruit production and ranking fourth in overall food production, is bananas, which are grown in over 130 countries. After harvest, almost 60% of banana biomass is left as waste. Worldwide, about 114.08 million metric tons of banana waste-loss are produced, leading to environmental problems such as the excessive emission of greenhouse gases [30]. Banana peels are a widespread and significant resource for bio-fillers, enabling the production of eco-friendly and biodegradable composites.

To demonstrate the novelty of our research, we conducted an extensive search of the academic literature using the Web of Science (WOS) and Scopus database using the keywords PLA, orange biochar, and banana biochar, and the results indicated only one article by Kim *et al.* [31] where developed PLA composites were reinforced with calcium-crosslinked orange peel biochar (CC-OPB). The results indicated significantly improved thermal and flame-retardant properties. Also, we found very few articles where the fillers used for PLA composites were orange or banana peels but with untreated thermal properties. For example, Sambudi *et al.* [32] conducted research on the incorporation of fine orange peel powder (OPP) (0, 10, 20, 40, and 60 wt.%) into PLA solution. The study found that adding OPP decreased tensile strength and Young’s modulus but increased elongation at low loadings. The hydrophilic OPP characteristics modified the surface hydrophobicity of the PLA, resulting in a rough surface and low surface energy. In another research, Koutoulis *et al.* [28] studied the influence of citrus peel powders on the physicochemical and biochemical properties of PLA films. The results showed that adding citrus powders to PLA was more efficient than the control films, resulting in high antioxidant capacity, phenolic content, lightness, elastic Young’s modulus (257.95–175.38 MPa), oxygen transmission rate, elongation at break (183.06–135.95%), and tensile strength (6.15–4.56 MPa). Kumar *et al.* [33] studied the impact of banana fillers on the interlocking strength and modulus of composites. The authors found that a small amount of banana peel fillers, up to 5.0 wt.%, significantly improved the mechanical features of the composites. However, excessive filler content led to agglomeration, reducing overall performance. The study found that samples with filler content lower than 5.0 wt.% showed superior wear resistance and reduced friction coefficients. Komal *et al.* [34] developed biocomposites with 20 wt.% banana fiber and polylactic acid to improve their mechanical properties. Three processing techniques were employed: direct injection molding (DIM), extrusion injection molding (EIM), and extrusion compression molding (ECM). The developed biocomposites exhibited significant enhancements in tensile and flexural characteristics, dynamic properties, and crystallinity. PLA/banana fiber composites were successfully fabricated by Nguyen and Nguyen [35] with different banana fiber contents: 10%, 20%, and 30 wt.%. Biocomposites with 20% fiber by weight showed better mechanical properties than the other contents. Therefore, to fill the gap, in our study, we investigated the impact of temperature on the conversion of banana and orange peels in the thermal decomposition process, the thermal conditions for obtaining a maximum amount of biochar, and the use of the obtained biochar as filler for PLA composites. The biomass waste was characterized by determining the composition by compound classes and thermogravimetric analysis (TGA), and the PLA composites containing 1, 2, and 3 wt.% BC were also characterized by tensile straight, TGA, differential scanning calorimetry (DSC), SEM, and atomic force microscopy (AFM).

## 2. Materials and Methods

### 2.1. Materials

Banana and orange peels were obtained from fruits purchased from local supermarkets (Ploiesti, Romania).

#### 2.1.1. Preparation of the Biomass

Raw banana peel and orange peel waste were washed with distilled water, then oven-dried at 50 °C for 24 h and cut into smaller pieces. The resulting dried peel was finely crushed with a GRINDOMIX GM 200 knife mill, manufactured by Retsch GmbH (Haan, Germany), and sieved to a powder with a particle size ≤ 200 µm [10].

#### 2.1.2. PLA Sample Preparation

Three composites of blends containing polylactic acid (from Nature Works LLC under the brand name Ingeo^®^, Tokyo, Japan), plasticizer diacetine (purchased from Sigma Aldrich, St. Louis, MO, USA), and oregano oil (from local natural products shop) were obtained by solubilization in chloroform at a temperature of 45 °C at 60 rpm and mixed for 30 min. The resulting mixture was poured into Petri dishes to obtain the polymer sample by removing the solvent by evaporation.

### 2.2. Biomass and PLA Composite Characterization

#### 2.2.1. Determination of Water Content

The analysis was carried out by weighing a certain amount of sample and drying it, repeatedly, in an oven at 103 °C until achieving constant mass. At intervals of 4–5 h, the sample vial was removed from the oven, cooled in a desiccator for 30 min, and then weighed on an analytical balance. The drying, cooling, and weighing operations were repeated until the difference between two successive weighing results was not greater than 0.004 g, and the mass loss was reported to 100 g of the initial sample.

#### 2.2.2. Determination of Extractables in Ethanol

The removal of extractables, such as waxes, fats, resins, gums, sterols, flavonoids, terpenes, etc., was achieved by extraction with ethanol in a Soxhlet for 16 h [36]. The ethanol extractables content was determined by reference to the initial sample.

#### 2.2.3. Determination of Lignin Content

The lignin content of banana and orange peel samples was determined according to Carrier *et al.* [37] and Standard E-1721, adapted [38]. According to this method, lignin (known as “Klason lignin”) was precipitated in a 72% sulfuric acid solution. Given the low lignin content of these biomasses, we treated 10 g of peels obtained after removing the extractables with 150 mL of 72% H_2_SO_4_ solution for 2 h at room temperature to hydrolyze and solubilize carbohydrates. We then diluted the sample with water to reduce the sulfuric acid concentration to 3% gr. and heated it at reflux for 4 h. The obtained lignin was filtered, washed with hot water until reaching a neutral pH, and then dried and weighed. The lignin content was determined by reference to the initial sample.

#### 2.2.4. Determination of Carbohydrate Content

The carbohydrate content of banana and orange peel samples was determined according to Standard E-1721, adapted. Thus, after lignin precipitation, the content of hemicellulose, cellulose, and other carbohydrates such as pectin, sugars, etc., represented the difference between the weight of the biomass sample without extractables and that of lignin.

#### 2.2.5. Determination of Ash Content

Ash determination was performed according to Standard Test Method for Ash in Biomass [39], ASTM E1755-01(2020). A crucible was cleaned and placed in a muffle furnace to burn any fuels. It was then cooled and placed in a desiccator. The weighted mass of the sample was placed in the crucible. The crucible with the sample dried at 105 °C was placed in an oven at 575 °C for 5 h, after which the crucible was cooled and weighed. The difference in weight, expressed as a percentage of the mass of the residue remaining after calcination, represented the ash content of the sample. The result was reported to the mass of the sample dried at 105 °C.

#### 2.2.6. Fourier Transform Infrared Spectroscopy (FT-IR)

To perform FTIR analysis, Shimadzu IRTRACER-100, Kyoto, Japan, was used in the 4000–400 cm^−1^ region with a resolution setting of 4 cm^−1^.

#### 2.2.7. Thermal Properties

Thermogravimetric analysis assessed the thermal stability of biomass and biochar using the thermogravimetric/derivative apparatus TGA/DTG (TGA 2 Star System Mettler Toledo, Zurich, Switzerland). Our study’s methodology involved increasing the temperature in a controlled nitrogen atmosphere from 25 to 800 °C at a rate of 5 to 30 °C per minute [10]. Differential scanning calorimetric (DSC) determination was performed with a DSC 3+ Star system from METTLER TOLEDO (Leicester, UK), under a N_2_ environment at 10 °C/min between 25 °C and 200 °C.

Proximate analysis was used to measure the amount of ash, moisture, fixed carbon, and volatile matter in peel powder according to the method reported by Reza *et al.* [40].

#### 2.2.8. Mechanical Properties

Tensile strength was determined on a Lloyds Instron Universal Analyzer (Lloyd Instruments, Ameteklns, West Sussex, UK), controlled by Nexygen software (version 4.0). The tests consisted of applying an increasing axial force (the loading force was 5 N) until rupture, at room temperature, according to the standard procedure EN ISO 527-3:2018 [41]. The specimens used in the tensile test had a rectangular section with 3 mm thickness, 4 mm width, and 40 mm height, presenting a calibrated portion and two ends for clamping in devices (3× 25 × 25 mm). Based on the stress–strain curves, the elongation at break (ɛ), tensile strength (UTS), and Young’s modulus (E) of each foil were calculated.

#### 2.2.9. Surface Analysis Equipment

The microstructural characteristics of the samples were determined using scanning electron microscopy using a SEM Inspect™ microscope manufactured by FEI, Hillsboro, OR, USA. The electron beam was accelerated to 30 kV under high vacuum to obtain secondary electron images. The surface topography of the samples was investigated by scanning atomic force microscopy (AFM) performed with a JEOL JSPM 4210 device, produced by JEOL, Japan, Tokyo. The scanned area was 20 μm × 20 μm, and the images obtained were analyzed with the specialized software Jeol WIN SPM 2.0 produced by JEOL, Japan, Tokyo. The surface roughness was measured using the Ra and Rq parameters according to the literature data [42].

#### 2.2.10. Rheological Properties

The prepared composites were subjected to rheological analysis to evaluate the effect of process parameters on the torque. The processing parameters were temperature, rotation speed, and time. The torque was studied from the mixing process. The tests were carried out in a Brabender Plastograph (Brabender^®^ GmbH & Co. KG, Duisburg, Germany) at a temperature of 180 °C for 6 min and at a speed of 60 rpm under a nitrogen atmosphere.

## 3. Results and Discussion

### 3.1. Characterization of Biomass Waste

#### 3.1.1. Analysis by Classes of Chemical Compounds of Biomass Waste Samples

Table 1 displays the analysis by classes of chemical compounds of orange and banana peel waste. Moisture content significantly impacts biomass feedstock processing. According to Ahmed *et al.* [43], the biomass with a water content below 15% is preferred for pyrolysis conversion. As can be seen in Table 1, both samples have low moisture contents of 2.5% for the banana peel and 3.26% the orange peel; therefore, they are suitable for the pyrolysis procedure. The high volatile content of 70.8% for banana peel and 75.34% for orange peel implies that during pyrolytic conversion, the biomass waste can be decomposed [44]. Banana peels had a high ash content (5.5%) compared to orange peels (2.21%). The fixed carbon content primarily represents the energy contained in the carbon–carbon bonds that contribute to the synthesis of biochar [45]. The fixed carbon concentration was found to range between 19.76 and 21.2%. Since lignin is the main precursor for biochar obtaining, banana peels had the highest fixed carbon concentration (21.2%), followed by orange peels (19.76%) [46]. The high volatile matter content and low ash content of fixed carbon are necessary for the surface, structure, and textural properties of the material to develop well, according to various research [43,46]. Thus, biomass waste samples have significant potential to produce large amounts of biochar.

#### 3.1.2. Thermogravimetric Profile of the Biomass Waste Samples

The literature indicates that the orange peel contains 23% sugar, 22% cellulose, 25% pectin, and 11% hemicellulose [47]. Meanwhile, the banana peel contains carbon-rich organic compounds such as cellulose (7.6–9.6%), hemicellulose (6.4–9.4%), pectin (10–21%), lignin (6–12%), chlorophyll pigments, and some other low-molecular-weight compounds (starch 0.78%, raw fiber 11.95%, crude protein 4.77%; calcium 0.36%, phosphorus 0.23%, lipids 1.15%, zinc 0.17%, and ash 1.71%) [30]

**Figure 1:**
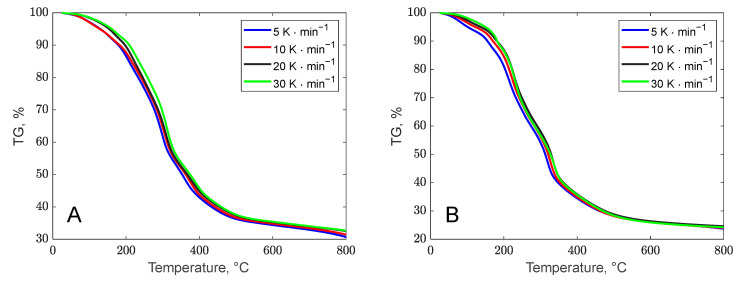
Different heating rates TG curves for banana (**A**) and orange (**B**) peels.

**Figure 2:**
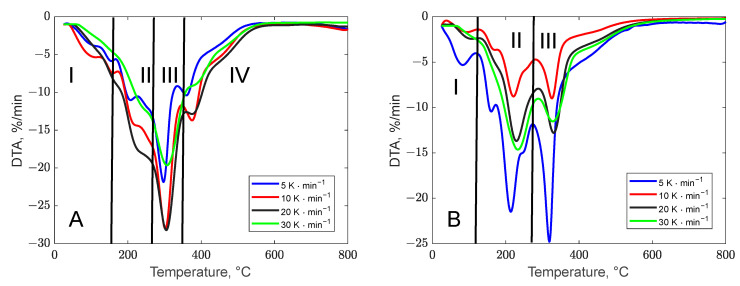
Different heating rates—DTG curves for banana (**A**) and orange (**B**) peel.

The thermal stability of biomass waste was assessed through thermogravimetric analysis. The thermal decomposition behavior of biomass residues is illustrated in Figure 1 via TG curves and in Figure 2 via DTG curves, for both banana peel (A) and orange peel (B), and the representations are in agreement with other literature studies [48,49]. Additionally, Table 2 provides the key peak temperatures and the residue amounts post decomposition. Although the decomposition pathways of the two biomasses appear similar at first glance, certain distinctions can be observed from the DTG curves. The degradation of residue samples was examined over the temperature range of 25 to 800 °C at varying heating rates (5, 10, 20, and 30 °C/min).

Region I of these curves corresponds to the release of moisture from the biomass structure. The weight loss recorded in this region for each biomass sample is approximately 5 to 8% of the initial weight. With increasing heating rates, the weight loss values for banana peel were observed to be 5.82%/min, 7.41%/min, 8.06%/min, and 4.58%/min at rates of 5, 10, 20, and 30 °C/min, respectively, while for orange peel, the values were 5.31%/min, 1.84%/min, 2.43%/min, and 2.41%/min under the same conditions.

The second degradation phase, attributed to devolatilization, varies slightly due to differences in the composition of the samples. Banana and orange peels, primarily composed of carbohydrates like disaccharides and starch, displayed unique thermal degradation behaviors, as indicated by the DTG curves.

Banana peel exhibited four degradation peaks on the TGA curve. The decomposition of compounds with low molecular mass occurred within the temperature range of 25–180 °C. The breakdown of hemicellulose structure was identified to occur between 220 and 344 °C. The third stage with a peak decomposition temperature of 306 °C indicated the degradation of cellulose and lignin [50]. The final phase involved the thermal degradation of lignin, which, due to its complicated structure consisting of aromatic rings, occurs within the temperature 360 °C to 800 °C [51].

The devolatilization degradation of orange peel occurred in two distinct stages (regions II and III), highlighting the heterogeneous nature of this biomass. This process took place within a temperature range of 140–400 °C, aligning with previously reported findings reported by Singh *et al.* [52]. The initial peak, observed between 150 and 260 °C with a maximum around 200 °C and a weight loss of 24%, is attributed to the decomposition of simple carbohydrates such as fructose, glucose, and sucrose. The second peak, ranging from 280 to 400 °C with a maximum at 320 °C and a weight loss of approximately 25%, corresponds to cellulose degradation. Additionally, a broad peak occurring at temperatures above 400 °C is associated with lignin degradation, which spans a wide temperature range. These characteristics align with findings reported in the literature [53]. The decomposition steps were more distinctly highlighted at slower heating rates (5 and 10 °C/min), while at faster rates (20 and 30 °C/min), the peaks became less defined. A similar trend was observed in the decomposition behavior of orange peel, as illustrated in Figure 1B.

#### 3.1.3. Thermal Decomposition Kinetic Modeling

The study of solid-state kinetics can be conducted through thermal analysis by monitoring variations in the properties of a sample as it undergoes heating or is kept at a steady temperature. In our research, reaction kinetics were examined using thermal data acquired under non-isothermal conditions with a uniform heating rate. The decomposition of biomass residues leads to the production of volatile compounds (gases) and solid compounds (char), and this decomposition process can be described by the following overall transformation [54]:

(1) $$\mathrm{Biomass}\left(s\right)\to \;\mathrm{Char}\left(s\right)+\mathrm{Volatiles}\left(g\right)$$

The decomposition of solid samples under isothermal conditions is typically represented through mathematical or kinetic models that describe the rate of decomposition as a function of temperature and time. These expressions often incorporate reaction rate equations and parameters such as activation energy, pre-exponential factor, and the degree of conversion, such as the following:

(2) $$\frac{d\alpha }{dt}=k\cdot f\left(\alpha \right)$$

The rate of weight loss or conversion during decomposition is governed by the Equation (2), where k represents the temperature-dependent decomposition rate constant, and f(α) is a function defining the relationship with the extent of conversion α.

Equation (3) can be used to calculate the conversion or weight loss rate:

(3) $$\alpha =\frac{m_{0}-m_{t}}{m_{0}-m_{\infty }}$$

where the initial weight $m_{0}$, the weight at a specific time $m_{t}$, and the final mass $m_{\infty }$ of the sample can be used to determine the conversion or weight loss rate.

In non-isothermal TGA experiments, the heating rate β can be defined as a function of time and temperature.

(4) $$\frac{d\alpha }{dt}=\frac{d\alpha }{dT}\left(\frac{dT}{dt}\right)=\beta \frac{d\alpha }{dT};\;\;\;\beta =\frac{dT}{dt}$$

Assuming the Arrhenius representation of the decomposition rate constant, the time dependence of conversion in Equation (4) can be described as follows:

(5) $$\frac{d\alpha }{dT}=\frac{k_{0}}{\beta }e^{-\frac{E_{a}}{R\cdot T}}f\left(\alpha \right)$$

The methods described in the literature for determining kinetic parameters from TG data rely on Equation (5) in its original or integral form (differential and integral approaches). These methods commonly utilize graphical representation of the data to quickly evaluate the form of f(α) and calculate the kinetic parameters $E_{a}$ and $k_{0}$. The integral form of Equation (5) is expressed as follows:

(6) $$g\left(\alpha \right)=\int_{0}^{\alpha }\frac{d\alpha }{f\left(\alpha \right)}=\frac{k_{0}}{\beta }\int_{T_{0}}^{T}e^{-\frac{E_{a}}{R\cdot T}}dT$$

in which $T_{0}$ is the initial temperature.

Different kinetic model functions f(α) and their corresponding $g\left(\alpha \right)$ are usually employed for the solid-state reactions, the most important among them being presented in Table 3.

#### 3.1.4. Model-Free Methods

Various model-free approaches have been introduced into the existing literature by Friedman [56] (Friedman method), Ozawa [57] and Flynn and Wall [58] (Flynn–Wall–Ozawa/FWO method), and Kissinger [59] (Kissinger–Akahira–Sunose/KAS method) as well as the Starink methods [60] and Vyazovkin method [61,62]. These rely on an isoconversional approach, where the conversion is considered constant, and the degradation rate is assumed to depend solely on the temperature.

The Kissinger–Akahira–Sunose (KAS) method is widely employed for determining kinetic parameters. Its general equation is expressed as follows [40]:

(7) $$\ln\left(\frac{\beta }{T_{m}^{2}}\right)=-\frac{E}{R}\left(\frac{1}{T_{m}}\right)-\ln\left[\left(\frac{E}{A\cdot R}\right)\int_{0}^{\alpha }\frac{d\alpha }{f\left(\alpha \right)}\right]$$

where T_m is the temperature at maximum decomposition rate. By plotting $\ln\left(\frac{\beta }{T_{m}^{2}}\right)$ in respect to $\frac{1}{T_{m}}$ and evaluation of the slope, one can calculate the apparent activation energy.

The Starink method refines the equations proposed by the Flynn–Wall–Ozawa (FWO) and Kissinger–Akahira–Sunose (KAS) methods, presenting them in a generalized form that enhances their accuracy. This approach is particularly useful for determining the apparent activation energy with improved precision. According to Starink’s work, the derived equation improves accuracy by an order of magnitude compared to the traditional isoconversional methods [63]:

(8) $$\ln\left(\frac{\beta }{T^{1.92}}\right)=const-1.0008\left(\frac{E_{\alpha }}{R\cdot T_{\alpha }}\right)$$

The Friedman method, a widely utilized isoconversional approach, involves applying the natural logarithm to both sides of the differential kinetic equation. This transformation yields a linearized form that facilitates the determination of the apparent activation energy at specific conversion levels [49]:

(9) $$\ln{\left(\frac{d\alpha }{dt}\right)}_{\alpha }=\ln\left[\beta {\left(\frac{d\alpha }{dT}\right)}_{\alpha }\right]=\ln\left({\left(k_{0}\right)}_{\alpha }f\left(\alpha \right)\right)-\frac{E_{\alpha }}{R\cdot T}$$

In this method, the reaction model function $f\left(\alpha \right)$ is assumed to be temperature-independent, meaning that the temperature influence is entirely captured by the degradation process, which correlates directly with the mass loss rate. To evaluate the activation energy, the logarithm of the conversion rate, $\ln\left(\frac{d\alpha }{dt}\right)$, is plotted against the reciprocal temperature $\frac{1}{T}$ at constant levels of conversion. The plot typically yields a straight line.

#### 3.1.5. Model-Fit Methods

The Coats–Redfern method is indeed a widely used integral approach for analyzing thermal decomposition kinetics. It incorporates the reaction mechanism into its formulation, providing a more detailed understanding of the process. The general form of the Coats–Redfern equation is as follows [55]:

(10) $$\ln\left(\frac{g\left(\alpha \right)}{T^{2}}\right)=\ln\left(\frac{A\cdot R}{\beta \cdot E_{\alpha }}\right)-\frac{E_{\alpha }}{R\cdot T}$$

From the linear plot, where the transformation of the data is represented as a function of $\frac{1}{T}$, the kinetic parameters ($E_{\alpha }$ and $k_{0}$) can be determined. To discern which reaction model aligns best with the data, the correlation coefficient value indicates a better match between the experimental data and the chosen kinetic model, allowing for effective discrimination among various evaluated models.

*The analysis of decomposition kinetics*

**Figure 3:**
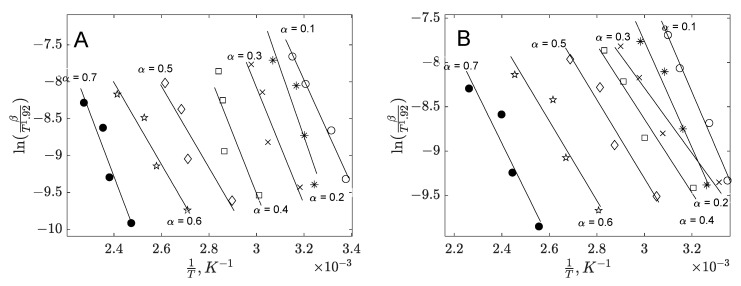
Starink plots for thermal decomposition of banana peel (**A**) and orange peel (**B**).

The kinetic analysis involved integrating the Arrhenius form of the decomposition rate constant with the law of mass action to determine the kinetic parameters for each decomposed biomass. This analysis utilized thermogravimetric (TG) data obtained at four distinct heating rates. To estimate the apparent activation energies, model-free methods were applied. Figure 3 presents the linear representation of the Starink plots for the two biomass residues.

**Single-Step Kinetic Model**

**Figure 4:**
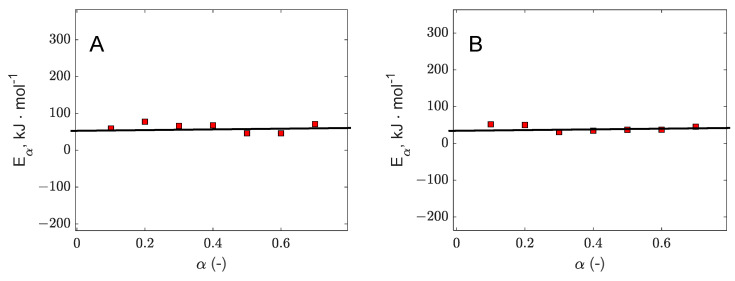
Apparent activation energies for different conversion values (banana peel—(**A**); orange peel—(**B**)).

When biomass decomposition is assumed to follow a single global reaction scheme, a single-step kinetic model serves as an effective approach to describing the decomposition process of biomass residues. This simplification assumes that the entire process can be represented by one dominant reaction, allowing for easier estimation of kinetic parameters and activation energy. Based on our findings, the conversion regime for applying the Starink single-step kinetic model was selected within the conversion range of 0.1–0.7 for both banana and orange peels. This range likely represents a stable and reliable segment for determining the kinetic parameters. In Figure 3 there are represented the parity diagrams for Starrink plots, whereas in Table 4 provides the corresponding values for the R^2^ coefficients, along with the slopes and intercepts derived from the linear regressions, offering a detailed quantitative assessment of the model’s fit and accuracy. The apparent activation energies $E_{\alpha }$ are plotted against the conversion α in Figure 4. For banana peel, the average value of $E_{\alpha }$ is 68 $kJ\cdot mol^{-1}$ and for orange peel 35 $kJ\cdot mol^{-1}$. The higher activation energy observed for the banana peels tested can be attributed to the much higher lignin content compared to orange peels. The literature data highlight higher activation energy values for lignin than for cellulose pyrolysis [64,65]. Additionally, the presence of certain minerals could contribute to the increased activation energy [41]. Comparable values for apparent activation energies have been reported in other studies [66,67] focusing on the decomposition of various bioresources.

***Multi-step kinetic model***

To determine the decomposition mechanism in each region shown in Figure 2, corresponding to the conversion ranges specified in Table 2, Coats–Redfern analysis was conducted using the theoretical forms of g(α) provided in Table 3. The left side of the corresponding equation was plotted against 1/T, and the degradation mechanism was evaluated based on the R^2^ coefficient for each region. In the case of banana peel thermal decomposition, Stage I with conversion values below 0.2 exhibited a three-dimensional diffusion mechanism. This behavior aligns with expectations, as it is attributed to the presence of low volatile compounds within the bioresidue structure [68]. For Stage II, taking place in a conversion range between 0.2 and 0.4, the decomposition mechanism corresponds to the contracting volume (R_3_) model. For Stages III and IV, at conversion values exceeding 0.4, the mechanism is identified as a third-order reaction (F3). This is likely attributed to the internal rearrangement of the carbon-rich chemical structure [69].

**Figure 5:**
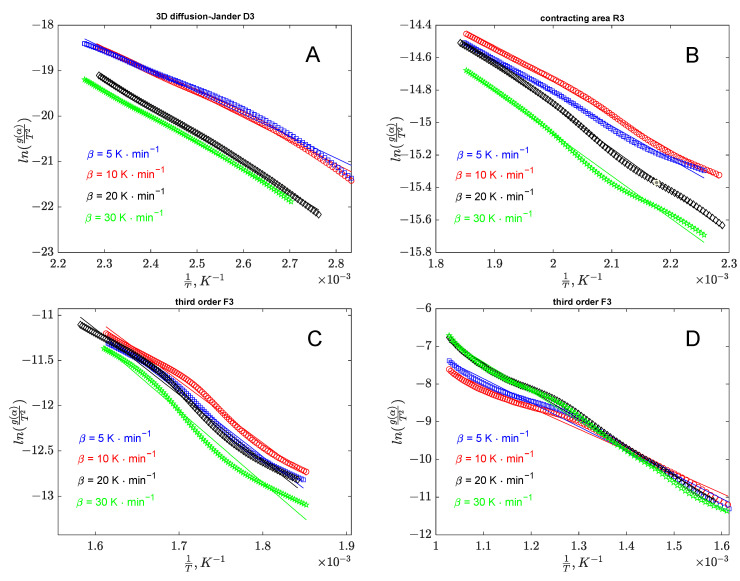
Parity diagrams from the analysis of banana peel degradation based on Coats–Redfern approach (**A**—region I; **B**—region II; **C**—region III; **D**—region IV).

**Figure 6:**
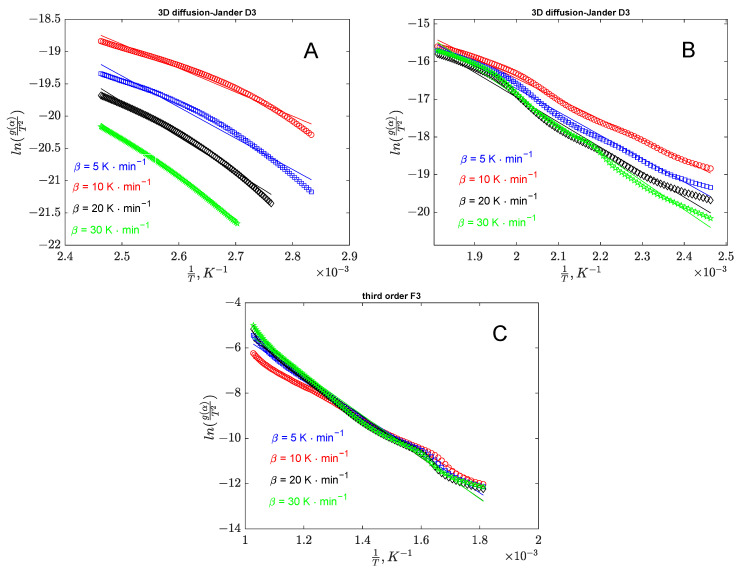
Parity diagrams from the analysis of orange peel degradation based on Coats–Redfern approach (**A**—region I; **B**—region II; **C**—region III).

The validation of the proposed models for each stage can be performed using the correlation coefficients presented in Table 5. Additionally, parity diagrams illustrated in Figure 5 for banana peel decomposition and Figure 6 for orange peel decomposition stages provide further support for the analysis.

### 3.2. FT-IR Analysis

**Figure 7:**
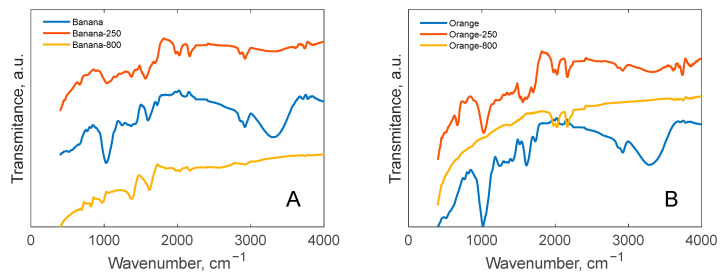
FT-IR spectra of samples (**A**—Banana; **B**—Orange).

The existence of vibrations from certain groups, including hydroxyl, aliphatic, carboxyl and carbonyl, ester and ether, aromatics, etc., has been shown by analysis of the FT-IR spectra of food waste biomass, biochar, and active coal samples. Around 3300 cm^−1^ is the typical peak of the hydroxyl zone, indicating the presence of alcohols, carboxylic acids, phenolics, and water. As the temperature rises, these signals become less intense, as shown in Figure 7 [70]. It is evident that this peak abruptly decreases in the orange, banana, and trash samples when heated to 250 °C.

The FT-IR spectrum showed bands corresponding to the stretching modes of C–H groups in the range of roughly 3100–2800 cm^−1^ as well as a peak at 1373 cm^−1^ attributed to C–H groups, which indicates the presence of methylene and methyl groups from cellulose and hemicellulose. The presence of ketones from hemicellulose was indicated by the C=O groups that were responsible for the peak at a wavenumber of 1730 cm^−1^ [46]. It is evident that aliphatic bonds are maintained at temperatures as low as 250 °C but vanish at temperatures as high as 800 °C. The banana at 800 degrees Celsius is the lone exception, though, where these connections still exist. The symmetric and asymmetric C–O stretching vibration mode is represented by the absorption peak seen at 1040 cm^−1^. Additionally, some carbohydrate functional groups (C–OH and C–OR groups) are indicated by this particular peak [71]. Additionally, the C-O bond from an ester or ether is represented by the peak at 1242 cm^−1^, whereas the C–H from aromatics is represented by the peak at 820 cm^−1^ [43]. In the samples made at high temperatures (800 °C), components like the C–O bond from ether or ester (i.e., 1247, 1240, 1246, and 1242 cm^−1^) lost their strengths. The presence of C=C vibrations in the aromatic skeleton are shown by the band at 1610 cm^−1^ [72].

### 3.3. Influence of Biochar on Thermal and Mechanical Properties of PLA Samples

Biomass fillers like natural fibers, starch, and cellulose can enhance the mechanical strength of PLA composites without compromising biodegradability. However, their high hydroxyl content can cause poor interfacial compatibility with hydrophobic PLA [11]. Biochar (BC) can address these filling deficiencies, as suggested by Qian *et al.* [73]. Thus, because based on the FT-IR analyses, the biochar obtained by pyrolysis at 800 °C of both biomass wastes did not exhibit peaks associated with polar functional groups such as hydroxyl, carboxyl, and carbonyl; it was evaluated as a filler for PLA samples (1, 2, and 3 wt.% biochar—PLA1, PLA2, and PLA3). The composites used in this study included 70 wt.% PLA, 2 wt.% oregano oil, from 1 to 3 wt.% biochar, and the rest diacetin.

#### 3.3.1. The Influence of Biochar Content on Thermal Properties

**Figure 8:**
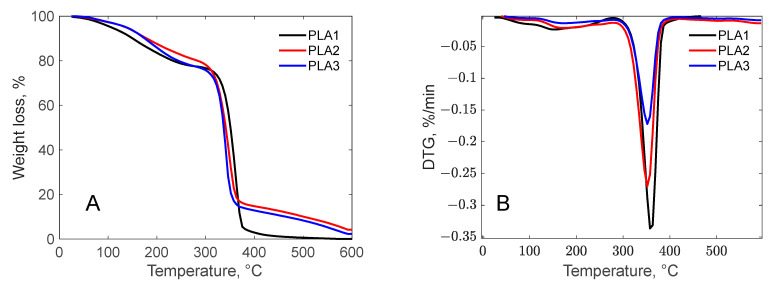
Thermogravimetric analysis of PLA composites (**A**—TGA curves; **B**—DTG curves).

The thermal stability of PLA composite containing 1 to 3 wt.% biochar is illustrated in Figure 8.

The degradation stage recorded in the range of 20–120 °C with a mass loss of about 2.77% for PLA-2 and PLA-3 samples and 4.25% for PLA-1, respectively, is attributed to moisture loss and indicates that the presence of a higher amount of biochar improves the hygroscopic properties. The two more obvious degradation stages appeared in the temperature ranges of 120–280 and 280–420 °C. The mass loss of about 16.27 and 19.10%, recorded between 120 and 208 °C with the maximum decomposition temperature around 208 °C, is associated with the loss of diacetin and oregano oil added as plasticizers. The thermal degradation of PLA was determined in the range of 280–420 °C, with a mass loss ranging from 16.27% for PLA-1 to 19.10% for PLA-3 sample. From the DTGA analysis, it was observed that, as the BC content increased, the maximum thermal degradation temperature moved towards lower temperatures from 366 °C for PLA-1 to 352 °C for PLA-2 and 345 °C for PLA-3 samples, respectively. These results are consistent with the studies developed by Xia *et al.* [11] and Qian *et al.* [73], who explained this phenomenon by the fact that the well-dispersed BC particles in the PLA matrix function as thermally conductive bridges, accelerating the heat transfer. The residue at 600 °C increases with increasing carbon content; thus, the ash generated by the PLA composite containing only 1% carbon has the lowest value of 8.26%, followed by the PLA-2% sample with 9.75 and PLA-3 with 10.95%. Zouari *et al.* [21] explained that the presence of BC led to the formation of a compact layer with high carbon content, and consequently, the residual amount increased.

**Figure 9:**
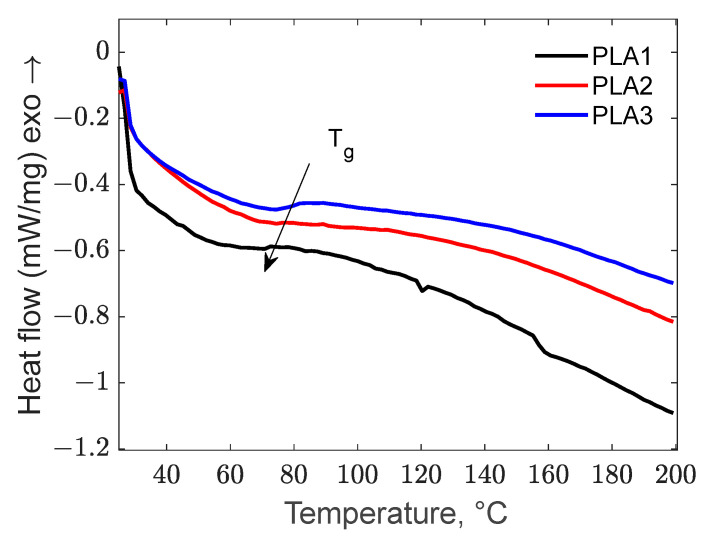
DSC thermogram of PLA composites.

The effect of the BC additive on the thermal transitions of PLA composites is presented in the DSC thermogram (Figure 9).

As can be observed, the addition of biochar to PLA has a positive effect on the Tg temperature, increasing as the added carbon increased, from 62.98 °C for 1 wt.% carbon to 75.35 °C for 2% and to 80.29 °C for 3 wt.%, respectively. This behavior is attributed to the hindered mobility of the PLA segment due to the addition of rigid biochar, thus requiring a higher heat absorption during the glass transition. This finding aligns with the research conducted by Qian *et al.* [73] and Papadopoulou *et al.* [7]. The PLA-1 sample exhibited two endothermic peaks at 120 and 158 °C, probably caused by the melting of amorphous structures present in low concentrations. Regarding the crystallinity degree of the samples, as can be seen in Figure 9, no sample presented an exothermic crystallization peak, indicating that the polymers are amorphous. This behavior was also observed by Papadopoulou *et al.* [7].

#### 3.3.2. Tensile Test Results

**Figure 10:**
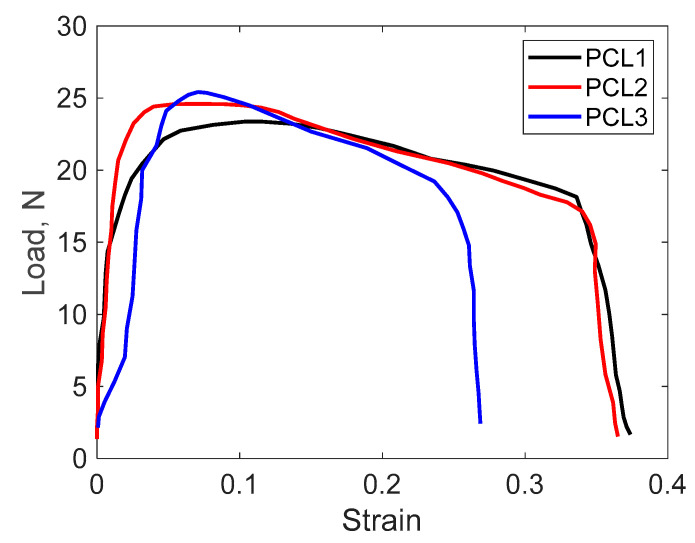
Deformation curve of samples at room temperature.

As can be seen in Table 6, the tensile strength of composites and Young’s modulus increased with the activated carbon content of the PLA samples, while the elongation decreased with the increase in the activated carbon content. Thus, the highest tensile strength and the lowest elongation were observed for the PCL3 formula (which had a content of 3 wt.% activated carbon). From Figure 10, it can be seen that the breaking force has close values for the three samples, with the increase in the activated carbon content of the PLA sample causing the reduction in the breaking force, and this reduction was more pronounced in sample PCL3, where the decrease in the unitary (strain) effort at breaking is more evident.

According to Figure 10, the elastic region (the initial linear portion) increases as the activated carbon content increases. Since activated carbon is rigid, its addition restricts the mobility of the polymer chains, resulting in increased stiffness and a steeper slope of the stress–strain curve. A good dispersion of activated carbon strengthens the matrix, improving load transfer and thus leading to higher tensile strength. Elongation at break typically decreases as the activated carbon content increases because the rigid activated carbon particles impede the movement of the polymer chains and act as crack initiation sites, making the composite more brittle, with a quicker drop in stress after the maximum point and consequently a lower elongation.

Similar results were obtained by Lekrine *et al.* [74] when preparing PLA biocomposites with fibers and biochar, with the tensile strength and elastic modulus being higher than those of the polymer.

#### 3.3.3. Surface Analysis Results

**Figure 11:**
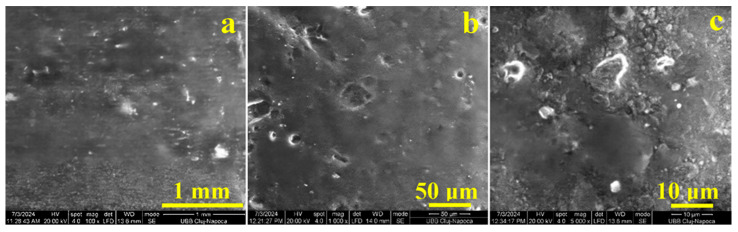
SEM images on the surface of samples PCL1, PCL2, and PCL3 at magnification of ×100, ×1000, and ×5000: (**a**) PLCX100l, (**b**) PLCX1000, and (**c**) PLCX5000.

The presence of diacetin in the composition of the PLA composite favored the advanced dispersion of the activated carbon powder, leading to the formation of an almost continuous matrix. Thus, the biochar was incorporated as a filler in this matrix of samples PCL1-3 (Figure 11). The surface of these samples was relatively smooth due to the addition of diacetin, but small microclusters of activated carbon aggregates were still visible, especially in the PCL3 sample. These aggregates were most likely formed due to the insufficient amount of plasticizer (diacetin), which determined a heterogeneous dispersion of the activated carbon filler.

**Figure 12:**
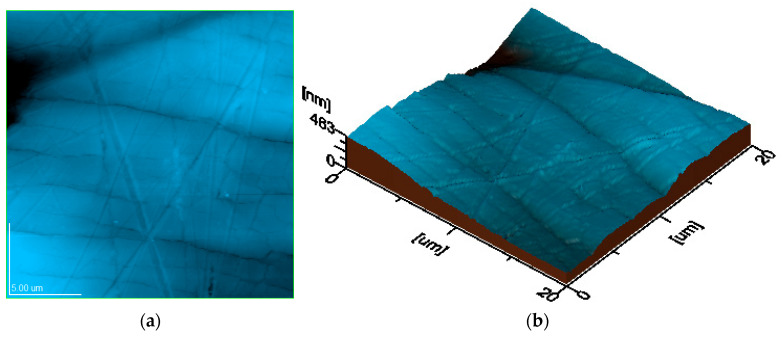
Topographic features of PCL1: (**a**) topographic image; (**b**) 3D image (scanned area 20 µm × 20 µm; Ra area 51.2 nm; Rq area 67.8 nm).

**Figure 13:**
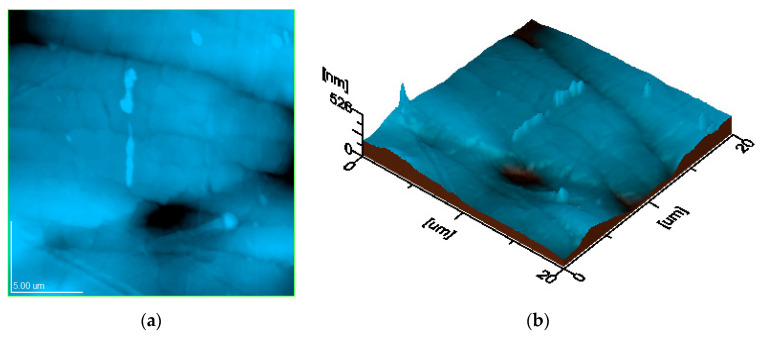
Topographic features of PCL2: (**a**) topographic image; (**b**) 3D image (scanned area 20 µm × 20 µm; Ra area 34.0 nm; Rq area 44.5 nm).

**Figure 14:**
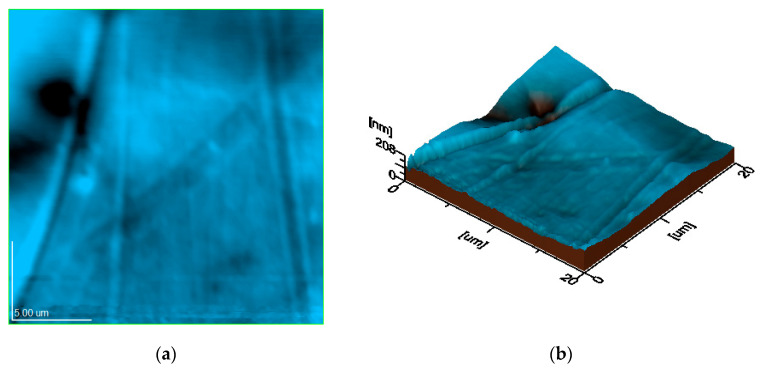
Topographic features of PCL3: (**a**) topographic image; (**b**) 3D image (scanned area 20 µm × 20 µm; Ra area 17.8 nm; Rq area 24.8 nm).

Studying the surface of the samples by atomic force microscopy (AFM) provided a more detailed view of the topographic features, revealing the dispersion of the filler nanoparticles, the variation in the composite thickness, as well as the finest microstructural units (Figure 12). The topographic image of the three samples highlights similar sizes of the biochar clusters for the three PLA composites, while the three-dimensional profile presents similar values of the topographic irregularities for the three samples and the reduced roughness of their surface, as shown in Figure 13 and Figure 14.

The literature data reveal that the submicron and nanoparticles distribution within surface topography directly influences its roughness and composite interaction with adjacent environment [75,76,77]. Thus, the biochar clusters incorporation in the polymer matrix ensures an optimal nanostructural distribution, which ensures proper surface uniformity and compactness. This relates to the bulk properties, which ensure a microstructural optimization of the composite with increasing biochar amount, a fact sustained by the increase in tensile strength. The topographic images in Figure 13 and Figure 14 reveal the progressive compactness of the filler particles regarding the polymer matrix induced by its increased amount. This leads to a relative microstructural stiffening, which causes a significant reduction in the elongation at the break. The optimal embedding of the filler particles within the polymer matrix ensures an elastic network comprising the rigid microstructural clusters, a fact explained by the progressive increase of the Young’s modulus with the biochar amount.

#### 3.3.4. The Influence of Biochar Content on Rheological Properties

**Figure 15:**
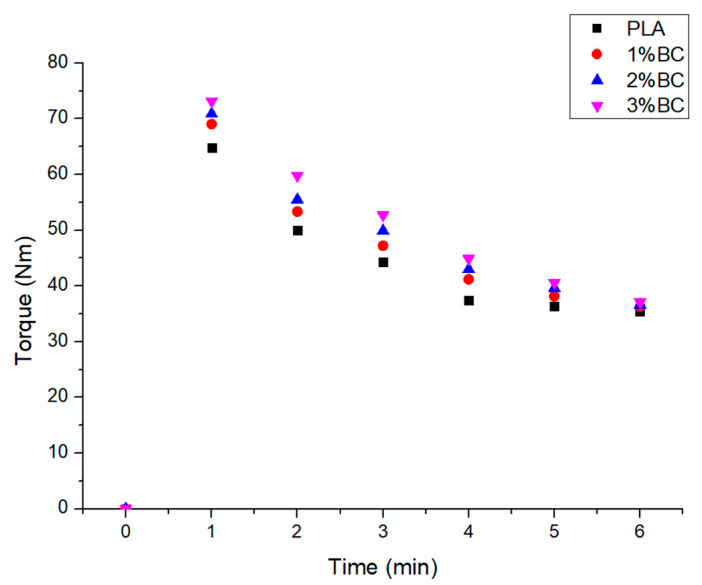
The plastograms of the PLA-based composites.

The plastograms of the PLA-based composites melted at a temperature of 180 °C, a screw rotation speed of 60 rpm, and a mixing time of 6 min are shown in Figure 15.

In this study, four samples with different biochar content were tested; thus, the PLA sample does not contain biochar, and the 1%BC, 2%BC, and 3%BC samples contain biochar at concentrations of 1%, 2%, and 3% wt, respectively. Initially, a sudden increase in torque was observed up to values over 60 Nm for all the tested samples, followed by a decrease with the duration of mixing. The decrease followed a steep slope in the first stage, and then, the decrease became slow and tended to attenuate after 5–6 min from the start of the melt processing. The increase in the biochar content determined an increase in torque, with the increase diminishing with the increase in the duration of the mixing process. Consequently, the selected biochar concentrations as well as the nature of the selected plasticizer determined a relatively small increase in torque compared to the initial PLA sample.

## 4. Conclusions

The present research investigated the incorporation of biochar derived from banana and orange peels into PLA, demonstrating its effects on thermal and mechanical characteristics. The thermal stability of biomass waste was evaluated by thermogravimetric analysis. The kinetic study was also performed through thermal analysis. Model-free methods were applied to estimate the apparent activation energies. For both banana peels and orange peels, the conversion regime for applying the one-step Starink kinetic model was selected in the conversion range of 0.1–0.7, which is considered a stable and reliable segment for determining kinetic parameters. The experimental data indicated that increased biochar content enhanced hygroscopic characteristics and shifted the maximum thermal degradation temperature to lower values. The Tg temperature increased from 62.98 °C for 1 wt.% to 80.29 °C for 3 wt.%. The surface examination indicated the presence of smooth samples with little microclusters of biochar aggregates, particularly in composites comprising 3 wt.% BC. Therefore, the research determined that incorporating biochar into PLA improves material performance while maintaining environmental sustainability. Future study should focus on evaluating long-term durability and biodegradability in real-world applications of the PLA composites.

## Figures and Tables

**Table 1:** Chemical composition of biomass waste samples.

**Biomass Waste**	Banana Peels	Orange Peels
Moisture, wt.%	2.50	3.26
Ethanol extractables, wt.%	14.35	12.57
Lignin, wt.%	11.68	1.21
Carbohydrates, wt.%	66.71	80.62
Ash, wt.%	4.72	2.29

**Table 2:** Different heating rates and TG and DTA characteristics.

Peel Type	Heating Rate, °C·min^−1^	Temperature of the Peaks, °C	$\frac{dW}{dt}$, %·min^−1^	Material Left, %
Banana	5	152 206.4 298.3 359.7	5.82 11.1 21.8 10.4	32.8
	10	155.1 216.4 301.3 375.2	7.41 14.2 28.5 13.7	33.4
	20	156.5 226.1 305.6 375.9	8.06 17.6 28.2 12.9	33.9
	30	157.3 237.9 306.8 382.3	4.58 12.0 19.6 9.03	34.1
Orange	5	80.3 215.4 319	5.31 21.43 24.72	25.1
	10	93.5 221.5 325.8	1.84 8.77 8.97	24.8
	20	105.2 232.1 332.3	2.43 13.7 12.7	25.3
	30	100.72 232.3 330.5	2.41 14.7 11.53	24.8

**Table 3:** Differential and integral shapes of kinetic models [49,55].

Mathematical Model		Differential Shape—f(α)	Integral Shape—g(α)
Power/Exponential			
Power law	P_2_	$2\cdot \alpha^{\frac{1}{2}}$	$\alpha^{\frac{1}{2}}$
	P_3_	$3\cdot \alpha^{\frac{2}{3}}$	$\alpha^{\frac{1}{3}}$
	P_4_	$4\cdot \alpha^{\frac{3}{4}}$	$\alpha^{\frac{1}{4}}$
Random nucleation and nuclei growth			
Avrami–Erofeev	A_2_	$2\cdot \left(1-\alpha \right)\cdot {\left[-\ln\left(1-\alpha \right)\right]}^{\frac{1}{2}}$	${\left[-\ln\left(1-\alpha \right)\right]}^{\frac{1}{2}}$
	A_3_	$3\cdot \left(1-\alpha \right)\cdot {\left[-\ln\left(1-\alpha \right)\right]}^{\frac{2}{3}}$	${\left[-\ln\left(1-\alpha \right)\right]}^{\frac{1}{3}}$
	A_4_	$4\cdot \left(1-\alpha \right)\cdot {\left[-\ln\left(1-\alpha \right)\right]}^{\frac{3}{4}}$	${\left[-\ln\left(1-\alpha \right)\right]}^{\frac{1}{4}}$
Contraction models (geometrical)			
Contracting area	R_2_	$2\cdot {\left(1-\alpha \right)}^{\frac{1}{2}}$	$1-{\left(1-\alpha \right)}^{\frac{1}{2}}$
Contracting volume	R_3_	$3\cdot {\left(1-\alpha \right)}^{\frac{1}{3}}$	$1-{\left(1-\alpha \right)}^{\frac{1}{3}}$
Diffusion models			
1D diffusion	D_1_	1/(2⋅α)	$\alpha^{2}$
2D diffusion	D_2_	$-\left[1/\ln\left(1-\alpha \right)\right]$	$\left[\left(1-\alpha \right)\cdot \ln\left(1-\alpha \right)\right]+\alpha$
3D diffusion (Jander)	D_3_	$\left[3\cdot {\left(1-\alpha \right)}^{\frac{2}{3}}\right]/\left[2\cdot \left(1-{\left(1-\alpha \right)}^{\frac{1}{3}}\right)\right]$	${\left[1-{\left(1-\alpha \right)}^{\frac{1}{3}}\right]}^{2}$
3D diffusion (Zhuravlev–Lesokhin–Tempelman)	D_4_	$3/\left[2\cdot \left({\left(1-\alpha \right)}^{\frac{1}{3}}-1\right)\right]$	$1-\left(2/3\right)\cdot \alpha -{\left(1-\alpha \right)}^{\frac{2}{3}}$
Reaction models			
Zero order	F_0_	1	α
First order	F_1_	(1−α)	$-\ln\left(1-\alpha \right)$
Second order	F_2_	${\left(1-\alpha \right)}^{2}$	$\left[1/(1-\alpha )\right]-1$
Third order	F_3_	${\left(1-\alpha \right)}^{3}$	$1/2\left[{\left(1-\alpha \right)}^{-2}-1\right]$

**Table 4:** Starink method results.

α	Banana Peel			Orange Peel		
	Slope	Intercept	R^2^	Slope	Intercept	R^2^
0.1	−7091.3	14.715	0.983	−6256.9	11.688	0.988
0.2	−9279.4	20.946	0.852	−6001.9	10.244	0.974
0.3	−7912.1	15.657	0.892	−3686.9	2.7736	0.947
0.4	−8138.6	14.901	0.744	−4163.7	3.8521	0.958
0.5	−5518.8	6.2879	0.877	−4439.9	4.0106	0.947
0.6	−5551.6	5.3139	0.945	−4488	3.0068	0.909
0.7	−8515.4	11.155	0.931	−5446.9	4.1624	0.907

**Table 5:** The results of the Coats–Redfern data analysis.

Banana Peel Kinetic Parameters			
Stage I	Stage II	Stage III	Stage IV
α<0.2 $E_{a}=45.8\frac{kJ}{mol}$ $k_{0}=3.93\cdot 10^{2}\;s^{-1}\;$ R^2^ = 0.994	0.2<α<0.4 $E_{a}=19.4\frac{kJ}{mol}$ $k_{0}=1.98\;s^{-1}\;$ R^2^ = 0.996	0.4<α<0.7 $E_{a}=60.8\frac{kJ}{mol}$ $k_{0}=\;3.54\cdot 10^{5}\;s^{-1}\;$ R^2^ = 0.989	0.7<α<1 $E_{a}=56.8\frac{kJ}{mol}$ $k_{0}=2.84\cdot 10^{5}\;s^{-1}\;$ R^2^ = 0.991
$f\left(\alpha \right)=D_{3}\;$−3D diffusion (Jander)	$f\left(\alpha \right)=R_{3}\;$–Contracting volume	$f\left(\alpha \right)=F_{3}\;$–Third order	$f\left(\alpha \right)=F_{3}\;$–Third order
**Orange Peel** **Kinetic Parameters**			
Stage I	Stage II	Stage III	
α<0.4 $E_{a}=42.1\frac{kJ}{mol}$ $k_{0}=4.99\cdot 10^{2}\;s^{-1}\;$ R^2^ = 0.984	0.4<α<0.7 $E_{a}=53.6\frac{kJ}{mol}$ $k_{0}=1.02\cdot 10^{4}s^{-1}\;$ R^2^ = 0.992	0.7<α<1 $E_{a}=70.6\frac{kJ}{mol}$ $k_{0}=5.64\cdot 10^{6}\;s^{-1}\;$ R^2^ = 0.993	
$f\left(\alpha \right)=D_{3}\;$−3D diffusion (Jander)	$f\left(\alpha \right)=D_{3}\;$−3D diffusion (Jander)	$f\left(\alpha \right)=F_{3}\;$–Third order	

**Table 6:** Tensile test results.

Sample	Tensile Strength (MPa)	Maximum Load (N)	Elongation at Break (mm)	Young’s Modulus (MPa)	Stress at Yield (MPa)
PCL1	2.621 ± 0.11	23.904 ± 1.21	14.179 ± 1.53	194.334 ± 8.26	1.341 ± 0.16
PCL2	2.957 ± 0.13	24.607 ± 2.43	10.666 ± 1.46	317.839 ± 10.1	0.591 ± 0.05
PCL3	3.432 ± 0.15	26.147 ± 2.65	7.240 ± 0.95	388.314 ± 12.4	2.088 ± 0.23

## Data Availability

The original contributions presented in this study are included in the article. Further inquiries can be directed to the corresponding author.
